# Birth position and obstetric anal sphincter injury: a population-based study of 113 000 spontaneous births

**DOI:** 10.1186/s12884-015-0689-7

**Published:** 2015-10-09

**Authors:** Charlotte Elvander, Mia Ahlberg, Li Thies-Lagergren, Sven Cnattingius, Olof Stephansson

**Affiliations:** 1 Department of Medicine, Division of Clinical Epidemiology, T2, Karolinska University Hospital, 171 76 Stockholm, Sweden; 2 The Department of Health Sciences, Faculty of Medicine, Lund University, Box 117, 221 00 Lund, Sweden

**Keywords:** OASIS, Anal sphincter tear, Upright delivery, Perineal tear

## Abstract

**Background:**

The association between birth position and obstetric anal sphincter injury (OASIS) in spontaneous vaginal deliveries is unclear.

**Methods:**

The study was based on the Stockholm-Gotland Obstetric Database (Sweden) from Jan 1^st^ 2008 to Oct 22^nd^ 2014 and included 113 279 singleton spontaneous vaginal births with no episiotomy. We studied risk of OASIS with respect to the following birth positions: a) sitting, b) lithotomy, c) lateral, d) standing on knees, e) birth seat, f) supine, g) squatting, h) standing and i) all fours. All analyses were stratified for parity. General linear models were used to calculate risk ratios (RR) adjusted for maternal, pregnancy and fetal characteristics.

**Results:**

The rates of OASIS among nulliparous women, parous women and women undergoing vaginal birth after a caesarean (VBAC) were 5.7 %, 1.3 % and 10.6 %, respectively. The rates varied by birth position: from 3.7 to 7.1 % in nulliparous women, 0.6 % to 2.6 % in parous women and 5.6 % to 18.2 % in women undergoing VBAC. Regardless of parity, the lowest rates were found among women giving birth in standing position and the highest rates among women birthing in the lithotomy position. Compared with sitting position, the lithotomy position involved an increased risk of OASIS among nulliparous (adjusted RR 1.17, 95 % CI 1.06-1.29) and parous women (adjusted RR 1.66, 95 % CI 1.35-2.05). Birth seat and squatting position involved an increased risk of OASIS among parous women (adjusted RR [95 % CI] 1.36 [1.03-1.80] and 2.16 [1.15-4.07], respectively). Independent risk factors for OASIS were maternal age, head circumference ≥35 cm, birth weight ≥4000 g, length of gestation ≥ 40 weeks, prolonged second stage of labour, non-occiput anterior presentation and oxytocin augmentation.

**Conclusions:**

Compared with sitting position, lateral position has a slightly protective effect in nulliparous women whilst an increased risk is noted among women in the lithotomy position, irrespective of parity. Squatting and birth seat position involve an increase in risk among parous women.

## Background

For centuries, pros and cons about different birth positions have been debated. For upright labour, several physiological advantages have been hypothesized and measured, such as effects of gravity, stronger uterine contractions, maternal satisfaction and feeling of control [1–3]. However, today the majority of women in the Western societies deliver in a dorsal, semi-recumbent/sitting or lithotomy position [4–7]. It is claimed that these positions enable the attending midwife or obstetrician to monitor the fetus and facilitate a hands-on approach to perineal management to lower the risk of obstetric anal sphincter injuries (OASIS) [8].

Obstetric anal sphincter injuries are related to long term maternal complications, such as anal incontinence [9–11], sexual dysfunction [12], pain [13] and a reduced quality of life [14–16]. The rates of OASIS have increased in Sweden and in many other high-income countries over the last decades [17–20]. It has been unclear whether such trends reflect differences in populations, differences in diagnosis and registration, or differences in management of delivery between and within countries [19, 21–23].

Previously reported non-modifiable risk factors for OASIS are primiparity [22, 24], previous caesarean delivery [22, 25], high birth weight [17, 22, 24, 26–29], occiput posterior position [26, 17–30, 31], prolonged second stage of labour [28, 29, 30] and increasing gestational age [24, 30]. Studies disagree on whether maternal age influences risk of OASIS [5, 22, 17]. Modifiable risk factors include instrumental delivery [20, 25, 28, 17–31, 32] augmentation [26] and midline episiotomy [32]. Inconsistent findings have also been reported for the association between birth position and OASIS [4, 5, 33, 34] Further, considering women’s right to make choices concerning birth position, it is also important to examine associations between birth position and risk of OASIS.

The aim of this study was to investigate the association between birth position and occurrence of OASIS in spontaneous vaginal deliveries using a large population-based cohort in Sweden.

## Methods

Data on mother, delivery and infant characteristics were obtained from the population-based Stockholm-Gotland Obstetric Database, based on the medical record system used for all maternity, delivery and postnatal care units in the region. Data from the medical record system is forwarded daily to the database, which contains information from 2008 and onwards. The database includes prospectively recorded standardized information from antenatal care, delivery (with partograph data) and the postpartum period for both mother and infant.

In Sweden, midwives are the primary caregivers during normal labour and birth. However, if complications occur, the midwife will notify an obstetrician. Swedish midwives diagnose and suture first and second degree perineal lacerations. If a midwife is unsure of the degree of the laceration or suspects a larger laceration (third or fourth degree), an obstetrician will be called upon for diagnosis and repair of the trauma.

### Study population

During the study period (from January 1^st^, 2008, through Oct 22^nd^, 2014), 175 522 singleton births were recorded. We excluded cesarean and vaginal instrumental births (*n* = 49 422), preterm births (≤36 completed weeks; *n* = 4 408), births that required an episiotomy (*n* = 4 508), births in non-cephalic presentations (*n* = 3 749), and stillbirths (*n* = 156). The final study cohort consisted of 113 279 live singleton term non-instrumental births.

### Exposures

The midwife reported birth position in the medical record system within the next hours after birth. The recording system allowed the midwife to choose between seven different positions which (translated from Swedish) were; *sitting, lithotomy, lateral, standing on knees, supine squatting,* and *standing*. If another birth position was used it was documented using free text under the heading of “Other:…”. All births on the *birth seat* and in *all fours* position were collected from this heading. Remaining births in free text were sorted into appropriate birth positions if possible. The residual 166 births that were not understandable were collapsed with 583 births that did not have any information on birth position into the birth position category “unknown”. There were no specific criteria for each position; the midwife freely decided which heading she/he judged to best describe the birth position used by the woman. The collected data does not reveal level of elevation of the head of the bed. In the present investigation, we used the following categories of birth position: a) sitting, b) lithotomy, c) lateral, d) standing on knees, e) birth seat, f) supine, g) squatting, h) standing or i) all fours. The most frequently used birth position was sitting, which was used as reference category.

### Outcome measurements

The primary outcome was obstetric anal sphincter injury (OASIS). Cases were defined by having both a Diagnosis Related Groups (DRG) code (i.e. a surgical code for suturing of OASIS) in combination with a definition by the midwife or the obstetrician by a checkbox in the obstetric electronic case notes and/or International Classification of Diseases, tenth revision (ICD-10) code for sphincter injury. The DRG code used was MBC33 and the following ICD-10 codes: O702, O702C, O702D, O702X (grade III which involve the anal sphincter complex) and O703 (grade IV, which extend to the rectal mucosa).

### Covariates

Selection of potential confounding factors were based on biological plausibility and on results from previous studies. From the partograph and birth records we obtained information on maternal age, induction of labor, use of epidural analgesia (EDA), oxytocin augmentation, duration of second stage, head circumference, presentation, birth weight, and hospital delivery unit. Data on maternal weight and height for calculation of body mass index (BMI) was retrieved from the records of the first antenatal visit, usually in the first trimester. Hospital delivery unit was included as a potential confounder to adjust for potential cluster effects. Gestational age was determined using the following hierarchy: a) date of embryo transfer (3.0 %), b) early second trimester ultrasound (95.2 %), c) date of last menstrual period reported at the first antenatal visit (1.8 %) and d) from a postnatal assessment (<1 %). Duration of second stage was determined using the recordings of exact times in the mother’s electronic case notes from full dilatation to delivery. This variable had 7 % missing among nulliparous women and 24 % missing among parous women.

### Statistical analyses

Nulliparous and parous women were analysed separately. Furthermore, women with vaginal birth after a primary caesarean section (VBAC) have shown to be at higher risk for OASIS than both nulliparous and other parous women [34]. Women with VBAC (*n* = 2 828) were therefore included in a separate group. Characteristics of the nulliparous women, parous women and women undergoing VBAC in relation to birth position were estimated and presented along with prevalence rates of OASIS in descriptive Tables 1 and 2. Due to low numbers of OASIS in each birth position group, further analyses were not performed on the VBAC group.

**Table 1:** Prevalence of maternal and neonatal characterstics in relation to birth position

Characteristics (%)		Total	Sitting	Lithotomy	Lateral	Knee	Birth seat	Supine	Squatting	Standing	All fours	Unknown
Nulliparous women	Number	44 942	17 294	11 906	5850	2660	4913	1214	342	243	235	285
	%											
	Maternal age >35 years	13	11	14	12	14	14	10	10	17	15	17
	Head circumference >35 cm	55	53	58	54	53	57	51	47	53	61	53
	Birthweight > 4000 g	11	10	14	10	10	11	10	10	12	14	12
	Length of second stage >90 min	45	43	58	27	35	39	36	39	37	40	45
	Gestational age >40 weeks	60	59	63	59	59	62	54	57	61	65	59
	Induction	15	14	19	14	10	11	14	13	7	8	18
	Epidural analgesia	57	57	68	53	43	51	50	56	42	53	55
	Non-occiput anterior presentation	3	2	4	2	1	2	2	3	3	2	14
	Oxytocin augmentation	61	60	79	52	43	50	50	54	40	51	58
Parous women	Number	65 486	27 054	7410	12 631	7478	5025	3687	469	858	427	447
	%											
	Maternal age >35 years	35	32	36	36	41	40	31	34	45	41	35
	Head circumference >35 cm	65	62	71	67	67	71	59	63	67	69	65
	Birthweight > 4000 g	22	20	29	22	23	24	16	22	24	28	24
	Length of second stage >90 min	7	5	16	5	4	7	3	9	7	8	6
	Gestational age >40 weeks	57	55	61	57	59	61	52	57	59	61	56
	Induction	14	14	21	14	9	9	15	11	10	11	33
	Epidural analgesia	26	26	39	25	19	28	18	28	16	20	24
	Non-occiput anterior presentation	4	3	7	4	2	4	3	4	2	3	20
	Oxytocin augmentation	26	25	48	23	17	23	20	25	16	19	22
Women undergoing VBAC	Number	2828	1054	864	387	149	225	77	21	18	16	17
	%											
	Maternal age >35 years	31	28	32	32	37	29	29	38	50	38	35
	Head circumference >35 cm	63	62	67	62	68	61	54	57	71	75	53
	Birthweight > 4000 g	18	19	18	18	18	17	12	19	22	50	0
	Length of second stage >90 min	39	36	50	34	23	42	29	40	12	31	29
	Gestational age >40 weeks	61	60	62	62	55	68	58	62	56	69	47
	Induction	15	14	18	13	11	11	10	24	6	6	18
	Epidural analgesia	62	60	71	58	53	59	56	67	33	69	47
	Non-occiput anterior presentation	2	2	3	2	0	2	1	5	6	6	18
	Oxytocin augmentation	58	55	71	49	41	51	48	57	28	63	53

**Table 2:** Prevalence (%) of OASIS in relation to birth position

		Total	Sitting	Lithotomy	Lateral	Knee	Birth seat	Supine	Squatting	Standing	All fours	Unknown
Nulliparous women	N of OASIS	2 548	931	850	250	126	279	58	15	9	13	17
	OASIS (%)	5.7	5.4	7.1	4.3	4.7	5.7	4.8	4.4	3.7	5.5	6.0
	3rd degree (%)	5.2	4.9	6.5	4.0	4.3	5.1	4.4	3.8	3.7	5.5	5.5
	4th degree (%)	0.5	0.5	0.6	0.3	0.4	0.6	0.4	0.6	0.0	0.0	0.5
Parous women	N of OASIS	859	323	194	124	70	82	37	10	5	6	8
	OASIS (%)	1.3	1.2	2.6	1.0	0.9	1.6	1.0	2.1	0.6	1.4	1.8
	3rd degree (%)	1.1	1.1	2.0	0.9	0.8	1.4	0.9	1.7	0.6	1.2	1.7
	4th degree (%)	0.2	0.1	0.6	0.1	0.1	0.2	0.1	0.4	0.0	0.2	0.1
Women undergoing VBAC	N of OASIS	301	100	106	32	16	27	13	3	1	1	2
	OASIS (%)	10.6	9.5	12.3	8.3	10.7	12.0	18.2	14.3	5.6	6.3	11.8
	3rd degree (%)	10.2	9.5	10.9	7.4	10.7	11.6	18.2	14.3	5.6	1.2	11.8
	4th degree (%)	0.6	0.0	1.4	1.0	0.0	0.4	0.0	0.0	0.0	0.2	0.0

We used general linear models to calculate risk ratios (RRs) and 95 % confidence intervals (CI) to estimate associations between OASIS and the following categorical variables: maternal age (<35 or ≥ 35 years), maternal height (<167 or ≥ 167 cm), BMI (<25 or ≥ 25 kg/m^2^), head circumference (<35 or ≥ 35 cm), birth weight (<4000 or ≥ 4000 g), gestational age (<40 or ≥ 40 weeks), EDA (no/yes), augmentation with (synthetic) oxytocin (no/yes), duration of second stage (<90 or ≥ 90 min), non-occiput anterior presentation (no/yes), induction (no/yes) hospital delivery units (A, B, C, D, E, F and G) and changes over time in years. In Sweden, dystocia during second stage refers to >2 h for the descending phase alternatively >3 h if epidural anesthesia is used, and >1 h for the pushing phase. There are no clear differences made with regards to parity. Due to this quite unclear description and definition of a long second stage in Sweden we decided to use 90 min for all women.

The covariates were then entered into the multivariable model, and variables that remained significant after adjustments for either nulliparous or parous women were presented.

The statistical software package SPSS 20.0 (SPSS Inc., Chicago, IL, USA) was used for all data analyses. The regional ethical committee at Karolinska Institutet, Stockholm, Sweden approved the study protocol (No. 2009/275-31 and No. 2012/365-32). According to the Personal Data Act in Sweden, informed consent does not have to be obtained from each individual in this type of registry based research.

## Results

**Fig. 1:**
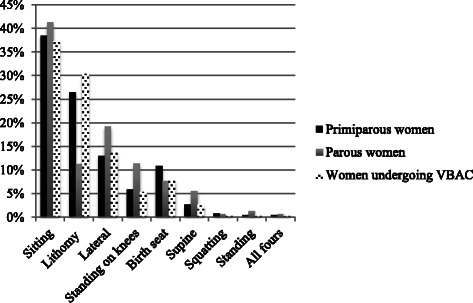
Rates of birthing positions (%) among nulliparous women, parous women and women undergoing VBAC, respectively

In total, we studied 44 942 births to nulliparous women, 65 486 births to parous women, and 2828 births to women undergoing VBAC. The most frequently used birthing position was sitting, both among nulliparous women (38.5 %), parous women (41.3 %) and women undergoing VBAC (37.3 %) (Fig. 1).

**Fig. 2:**
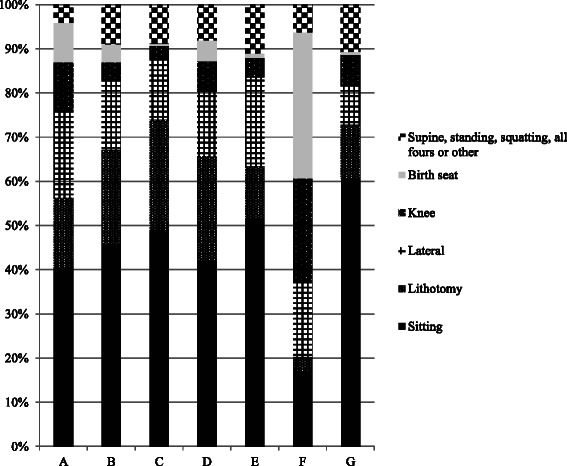
Rates (%) of birth positions used in relation to in-hospital birth unit (**a**-**g**)

There were large variations in practice of birth positions amongst the seven in-hospital delivery units. For instance, the rate of the sitting position varied from 17.0 to 60.7 % and the use of birth seat from 0.3 to 33.0 % (Fig. 2).

Table 1 shows maternal, neonatal and obstetrical characteristics of the nulliparous women, parous women and women undergoing VBAC in relation to birth position. Irrespective of parity, women giving birth in the lithotomy position were characterized by high rates of induction, EDA, oxytocin augmentation, long second stages, infants with large head circumferences, high birth weights and births of infants presenting in non-occiput anterior presentations.

The prevalence of OASIS among nulliparous women was 5.7 %, among parous women 1.3 % and among women undergoing VBAC 10.6 % (Table 2). The rate of OASIS varied by birth position: from 3.7 to 7.1 % in nulliparous women, 0.6 % to 2.6 % in parous women and 5.6 % to 18.2 % in women undergoing VBAC. The lowest rates were, regardless of parity, found among women giving birth in standing position.

Among nulliparous women and compared with the sitting position, the lithotomy position was associated with a modestly increased risk of OASIS in the adjusted analysis (Table 3). Only lateral position was associated with a reduced risk of OASIS in nulliparous women (adjusted RR 0.79, 95 % CI 0.68-0.92).

**Table 3:** Unadjusted and adjusted risk ratios and 95 % CI for OASIS among nulliparous and parous

	Nulliparous (*n* = 44 942)		Parous (*n* = 65 486)	
	Unadjusted risk ratio	Adjusted risk ratio*	Unadjusted risk ratio	Adjusted risk ratio*
	95 % CI	95 % CI	95 % CI	95 % CI
Total				
Sitting	1.00	1.00	1.00	1.00
Lithotomy	1.33 (1.21-1.46)	1.17 (1.06-1.29)	2.19 (1.83-2.62)	1.66 (1.35-2.05)
Lateral	0.79 (0.69-0.91)	0.79 (0.68-0.92)	0.82 (0.67-1.01)	0.84 (0.66-1.06)
Knee	0.88 (0.73-1.06)	0.88 (0.71-1.09)	0.78 (0.60-1.00)	0.81 (0.58-1.11)
Birth seat	1.05 (0.92-1.21)	1.05 (0.90-1.22)	1.37 (1.07-1.74)	1.36 (1.03-1.80)
Supine	0.89 (0.68-1.16)	0.91 (0.67-1.22)	0.84 (0.60-1.18)	0.84 (0.55-1.30)
Squatting	0.81 (0.49-1.36)	0.66 (0.36-1.24)	1.79 (0.95-3.35)	2.16 (1.15-4.07)
Standing	0.69 (0.36-1.33)	0.71 (0.34-1.49)	0.49 (0.20-1.18)	0.40 (0.10-1.61)
All four	1.03 (0.59-1.78)	1.12 (0.62-2.05)	1.18 (0.53-2.64)	0.95 (0.30-2.96)
Maternal age (years)				
<35	1.00	1.00	1.00	1.00
>35	1.25 (1.12-1.39)	1.21 (1.08-1.36)	1.13 (0.99-1.30)	1.12 (0.96-1.32)
Head circumference				
<35	1.00	1.00	1.00	1.00
>35	1.76 (1.62-1.92)	1.42 (1.29-1.57)	1.95 (1.65-2.31)	1.52 (1.23-1.88)
Birth weight				
<4000 g	1.00	1.00	1.00	1.00
>4000 g	2.16 (1.96-2.39)	1.69 (1.52-1.88)	2.54 (2.22-2.91)	1.86 (1.57-2.21)
Length of second stage				
<90 min	1.00	1.00	1.00	1.00
>90 min	1.36 (1.25-1.47)	1.15 (1.05-1.26)	2.44 (1.98-3.01)	1.93 (1.53-2.45)
Length of gestation (weeks)				
<40	1.00	1.00	1.00	1.00
>40	1.50 (1.38-1.64)	1.20 (1.09-1.31)	1.64 (1.42-1.87)	1.25 (1.04-1.49)
Epidural analgesia				
No	1.00	1.00	1.00	1.00
Yes	1.02 (0.94-1.10)	0.82 (0.74-0.90)	1.28 (1.11-1.47)	1.01 (0.88-1.24)
Non-occiput anterior presentation				
No	1.00	1.00	1.00	1.00
Yes	1.48 (1.20-1.82)	1.30 (1.01-1.68)	1.92 (1.47-2.51)	1.51 (1.10-2.05)
Oxytocin augmentation				
No	1.00	1.00	1.00	1.00
Yes	1.31 (1.21-1.42)	1.21 (1.09-1.35)	1.33 (1.15-1.53)	0.93 (0.78-1.12)

*Adjusted for all other variables in table + time in years + in-hospital delivery unit

Among parous women, the unadjusted analyses showed that lithotomy position was, compared with sitting position, associated with a more than two-fold increased risk of OASIS (Table 3). This increased risk was attenuated after adjustments. The birth seat position was associated with almost a 40 % increased risk of OASIS, and this increased risk was not attenuated after adjustments. Also in parous women, squatting position was not significantly associated with OASIS in the univariate analyses. In in the adjusted analysis, squatting position was associated with a more than two-fold increased risk of OASIS (Table 3). Other birth positions were not associated with risk of OASIS in parous women.

In multivariate analyses of nulliparous and parous women, we also found the following factors to be associated with increased risk of OASIS: large head circumference, high birth weight, prolonged second stage of labour, gestational age ≥40 weeks, and non-occiput anterior presentation (Table 3). High maternal age, oxytocin augmentation and hospital delivery unit was only associated with increased risk of OASIS in nulliparous women. Epidural analgesia had a protective effect in nulliparous women but was not significantly associated with OASIS in parous women. Maternal height, BMI and induction were not associated with increased risks of OASIS in the adjusted analysis (data not shown).

## Discussion

We found that the lithotomy position was associated with increased risk of OASIS, especially in parous women. The less frequently used birth seat and squatting position also implied an increased risk of OASIS in parous women. Compared with the reference category of sitting position, lateral position was associated with a slightly protective effect in nulliparous women.

Irrespective of parity, births in the lithotomy position had higher rates of OASIS but were more often accompanied by other risk factors for OASIS, such as high birth weight, large head circumference, non-occiput anterior presentation, oxytocin augmentation and a prolonged second stage of labour. These factors partly explained the association between lithotomy position and risk of OASIS. The increased risk that remained after adjustments supports findings from a Swedish observational cohort study [4]. Two additional studies have shown slightly increased risks of perineal trauma in lithotomy position, but these studies were not powered to specifically study birth position and risk of OASIS [35, 36]. Possible explanations of the association between lithotomy birth position and risk of OASIS may be that lithotomy position increases pressure sensations in the perineal area and decreases the woman’s ability to moderate the tempo of her own pushing efforts [35]. Further, the lithotomy position might increases the risk of OASIS by causing a greater pressure towards the sphincter during the expulsion of the infant compared with alternate positions.

We found a doubled risk of OASIS in squatting position among parous women. The increased risk in this position has been reported previously [4]. In contrast to others [5], we analysed birth seat position and squatting separately. Women in the squatting position lean forward more than women in the birth seat position who most often lean back into the arms of their partner sitting behind on a chair. Although an association between birth seat position and increase in second-degree tears previously has been reported [8], the association between birth seat and increased risk of OASIS in parous women found in our study is a novel and important finding. It is plausible that both the squatting position and the use of birth seat may lead to a too rapid expulsatory phase in parous women. This, in combination with challenging circumstances for the midwife to hands on protect the perineum may increase the risk of OASIS. Slowing the delivery of the infants’ head and supporting the perineum are interventions that have shown to lower the risk of OASIS [37].

The increased rate of OASIS among women undergoing VBAC may be explained by fetopelvic disproportion leading to a primary caesarean section possibly predisposing to OASIS at first vaginal delivery [25, 38]. We lacked information about indication for these women’s caesarean sections and if the birthing process in women undergoing VBAC differed from non-VBAC women.

The 6 % rate of OASIS among nulliparous women in our study population is reflected by the official rate reported by The National Board of Health and Welfare [39]. There is a significant difference in the Nordic countries in the incidence of OASIS [23]. It has been hypothesized that differences in delivery techniques and use of episiotomies may be one reason [23]. Although a Norweigan intervention study suggest that the obstetric practice and hands on practice makes a difference [37] another meta-analysis concluded that hands off (or poised) versus hands on showed no effect on third- and fourth-degree tears [40]. In Sweden, episiotomies are uncommon with a rate of <8 % among nulliparous women, while in Norway and Finland, corresponding rates are 23 % and 65 %, respectively [37, 41].

### Strengths and limitations

Major strengths of this study are the large sample size of births from all maternity and delivery units within a geographically defined area, and that data were obtained from prospectively recorded information in antenatal and obstetrical records. We were able to control for a number of possible confounders, including cluster variation between hospitals. The restriction of analyses to women with no episiotomy is a strength as it increases clarity although it limits generalizability. Lack of information on fundal pressure [26] and perineal protection is a limitation. Midwives may use various perineal management techniques [40] and such information was not systematically documented. Furthermore, we were unable to analyse the midwives’ experience and training, which could impact on outcome. However, all midwives at the hospital delivery units have received similar midwifery training even though the attitudes towards encouraging women to deliver in alternate positions might differ [8, 42].

This is an observational study, and the results cannot be causally interpreted. However, the findings are biologically plausible and have partially been supported in other studies [4, 8, 43].

## Conclusion

Compared with sitting position, lateral position has a slightly protective effect in nulliparous whilst an increased risk is noted among women in the lithotomy position, irrespective of parity. Births in the lithotomy position were accompanied by other risk factors for OASIS which partly explained the elevated risk. Squatting and birth seat position involved an increase in risk among parous women.
